# Influence of chemoradiation on the immune microenvironment of cervical cancer patients

**DOI:** 10.1007/s00066-022-02007-z

**Published:** 2022-10-17

**Authors:** J. M. Herter, M. Kiljan, S. Kunze, M. Reinscheid, O. Ibruli, J. Cai, L. Niu, I. Heßelmann, M. Trommer, G. S. Herter-Sprie, C. Köhler, S. Marnitz

**Affiliations:** 1 Department of Radiation Oncology, CyberKnife and Radiotherapy, University Hospital Cologne, Kerpener Str. 62, Geb. 3a, 50937 Cologne, Germany; 2 Center for Molecular Medicine Cologne, University Hospital of Cologne, Cologne, Germany; 3 Center for Integrated Oncology (CIO), University Hospital of Cologne, Cologne, Germany; 4 Department I of Internal Medicine, University Hospital Cologne, Cologne, Germany; 5 Women’s Clinic/Gynecology, Asklepios Clinic Altona, Hamburg, Germany

**Keywords:** Cervical cancer, Immune microenvironment, Chemoradiation, Immune checkpoint, Tumor microenvironment

## Abstract

**Purpose:**

Cervical cancer remains a leading cause of cancer death in women. While immunotherapy has shown great success in combating cancer, the value of immunotherapy in cervical cancer is still only beginning to be explored. Thus, we performed a prospective analysis of patient blood and tumor samples at the beginning and end of conventional chemoradiation to assess changes in the immune cell and immunoreceptor compartments, and investigate if and when the addition of immunotherapy could be beneficial.

**Methods:**

Patients with FIGO II–III cervical cancer receiving standard chemoradiation between January 2020 and December 2021 were included. We collected tumor and blood samples from patients before and at the end of therapy and analyzed immune cell composition and immune checkpoint receptor expression on both immune and tumor cells using multicolor flow cytometry.

**Results:**

In all, 34 patients were eligible in the study period; 22 could be included and analyzed in this study. We found that chemoradiation significantly reduces T cell numbers in both tumors and blood, but increases macrophage and neutrophil numbers in tumors. Furthermore, we found that the percentage of immune checkpoint receptor PD‑1 and TIGIT-expressing cells in tumors was significantly reduced at the end of therapy and that CD4 and CD8 memory T cell populations were altered by chemoradiation. In addition, we observed that while PD-L1 expression intensity was upregulated by chemoradiation on blood CD8 cells, PD-L1 expression frequency and the expression intensity of antigen-presenting molecule MHC‑I were significantly reduced on tumor cells.

**Conclusion:**

Our data demonstrate that chemoradiation significantly alters the immune cell composition of human cervical tumors and the expression of immune checkpoint receptors on both lymphocytes and tumor cells. As our results reveal that the percentage of PD‑1^+^ CD8 cells in the tumor as well as the frequency of PD-L1-expressing tumor cells were reduced at the end of therapy, neoadjuvant or simultaneous anti-PD‑1 or anti-PD-L1 treatment might provide better treatment efficiency in upcoming clinical studies.

**Supplementary Information:**

The online version of this article (10.1007/s00066-022-02007-z) contains supplementary material, which is available to authorized users.

## Introduction

Cervical cancer is the fourth most frequently occurring malignancy in women and among the leading cause of cancer death in women worldwide [1]. Primary chemoradiation is the established standard of care in patients with locally advanced and/or lymph node-positive cervical cancer. Several randomized trials demonstrated a significant benefit concerning local control, disease-free survival, and overall survival (OS) compared with radiation alone [2–7].

While improved radiation techniques provide acceptable local control rates, the high rate of distant metastases remains a challenge, causing a 5-year OS of merely 60% once the cancer spread to the parametrial space (stage IIB, 35% of all patients) and 20% for more advanced stages (17% of patients) [7]. According to the new International Federation of Gynaecology and Obstetrics (FIGO) classification [8], para-aortic metastases are no longer considered as distant metastases and provide a better prognosis for patients, but only 50% of these patients achieve long-term remission [9]. Furthermore, the OS for patients with other organ metastases remains particularly poor with a median survival of less than 1 year [10].

While the prognosis following treatment remains disappointingly low, the treatment toxicity of chemoradiation remains high, including hematologic, gastrointestinal (GI), and genitourinary (GU) toxicities. GI toxicity is a common problem for approximately one third of cervical patients undergoing concurrent chemoradiation and has an immense impact on the quality of life. High-grade anemia (7%), leukopenia (16%), and thrombocytopenia (2%) are also reported in patients. Of note, severe late toxicity has been reported even 3 years after completion of treatment in 35% of the patients [11]. While treatment escalation with adjuvant chemotherapy after chemoradiation did improve oncologic results, the cost was a significant simultaneous escalation of treatment toxicity to unacceptable levels [12].

In the last decade, the implementation of immunotherapy to treat different types of cancer has opened new avenues and demonstrated high rates of durable response in patients with recurrent and/or metastatic cervical cancer compared to conventional chemotherapy for metastatic diseases [13–17]. Interestingly, an increasing number of studies, mainly in lung cancer and melanoma, report a synergism between radiation therapy (RT) and immunotherapy [18, 19]. Recently, Herrera et al. confirmed this synergism for ovarian cancer [20]. In line with these findings, early studies of RT in cervical cancer patients have suggested a beneficial immunological effect [21, 22].

Immunotherapeutic strategies include targeting inhibitory receptors on T cells (most commonly programmed cell death protein‑1 [PD-1], or its ligand PD-L1 or cytotoxic T‑lymphocyte associated protein‑4 [CTLA-4]) in order to block an inhibitory feedback loop causing T cells to become dysfunctional. These therapies thus aim to reinvigorate the tumor immunity of the adaptive immune system causing effective tumor lysis by host T cells. While these regimens have shown great oncologic success, treatments are usually well tolerated alone [23] and also cause a very limited additional toxicity in combination with RT [24] or even chemotherapy [25, 26].

Several trials have been initiated in the fields of primary chemoradiation and immunotherapy and/or maintenance treatment in cervical cancer patients [27–30]. While two clinical studies are currently investigating the effects of anti-PD‑1 (NCT03614949) as well as anti-PD-L1 and anti-CTLA‑4 (NCT03452332) in combination with SBRT for metastatic, persistent or recurrent cervical cancer, two studies are currently examining a possible benefit of added anti-PD‑1 to chemoradiation ([31] and NCT02635360). To date, there is encouraging phase I/II trial data on the effects of PD‑1 blockade in recurrent/metastatic human papillomavirus (HPV)-associated cervical malignancies. The study reported an overall response rate of 26% in 19 patients with cervical cancer [32], while another phase IB study using anti-PD‑1 in 24 women with PD-L1-positive cervical cancer reported a 17% overall response rate [33]. An ongoing phase II trial of pembrolizumab (NCT02628067) preliminarily suggested a 17% overall response rate among the first 47 patients [34]. While these studies were conducted in recurrent or metastatic disease and may thus constitute diseases immunologically different from the primary curative treatment setting, they indicate that patients with cervical cancer could in fact benefit from immunotherapy targeting PD‑1 as the response rates are comparable to early data on PD‑1 monotherapy in high tumor mutational burden small cell lung cancer (SCLC) [17]. However, the available data also indicate that combining anti-PD‑1 therapy with anti-CTLA‑4 might be required to increase treatment response rates ([17] reported doubling of response rates in the aforementioned group using combination treatment). Therefore, it seems plausible that the addition of immunotherapy to RT or chemoradiation could potentially improve treatment outcomes and, in case of substitution of chemotherapy by immunotherapy, reduce treatment toxicity. However, in contrast to the studies showing synergy of RT and immunotherapy for melanoma and lung cancer, the first clinical trials with immune checkpoint inhibitors and RT in head and neck cancer failed to show clinical benefits [35]. While the reason for these surprising results is not entirely understood, beside timing of immunotherapy in the context of RT and as choice of concomitant chemotherapy have been discussed, as well as PD‑1 upregulation rather than PD-L1 by chemoradiation, which might favor anti-PD‑1 instead of anti-PD-L1 therapy (ascopost.com/issues/august-10-2021/javelin-head-and-neck-100-trial-when-failure-seems-fatal-hope-is-not-lost/). Thus, it seems to be of clinical interest to investigate the impact of chemoradiation on PD-L1 and PD‑1 in cervical cancer patients especially in the context of upcoming studies. Furthermore, information on the alteration of these targets by standard chemoradiation could aid the decision about the timing of immunotherapy.

Here, we present a thorough analysis of the immune microenvironment as well as inhibitory receptors on adaptive immune cells in both blood and tumor samples of cervical cancer patients before initiation of chemoradiation as well as before the completion of chemoradiation.

## Materials and methods

### Patient samples

The present studies were reviewed and approved by the local ethics committee. The study was conducted according to the Declaration of Helsinki and informed consent was obtained from all subjects. Blood and cervical tumor samples from 22 human subjects treated at our department were collected between January 2020 and December 2021. Patients with an indication for primary chemoradiation for FIGO stage II–III cervical cancer were selected. We first attempted to retrieve ‘end’ tumor samples at the 5th afterloading (5.AL) as this was the latest time point possible. However, we routinely found almost no tumor left on clinical inspection, and thus moved the sample acquisition to the 1.AL. All samples analyzed in this study were obtained at the 1.AL.

### Immune cell isolation and analysis

Tumor cells and tumor-associated immune cells were isolated and analyzed as previously described [36]. Briefly, cervical tumor biopsies were stored in ice-cold DPBS w/Ca,Mg (Gibco) until further procedure. After dissociation of the tumor tissue with DNAse I (50 µg/ml, Sigma Aldrich) and Collagenase IV (100 U/ml, Invitrogen) in RPMI +10% FBS for 45 min at 37 °C, single-cell suspensions as well as blood samples were treated with ACK lysis (Lonza) and used directly for staining. Prior to surface marker staining, isolated cells were stained using Zombie NIR (Biolegend). All antibodies used were purchased from Biolegend: CD16-FITC (#302006), CD19-PerPC-Cy5.5 (#302230), PD-L1-PE (#329706), IgG2b-PE (#400314), CD56-PE-Dazzle 594 (#362544), MHC-I-PE-Cy7 (#311430), CD66b-APC (#305118), CD45-Alexa700 (#368514), PD-L2-BV421 (#329616), IgG2a-BV421 (#400260), CD3-BV510 (#317332), HLA-DR-BV605 (#307640), CD123-BV650 (#306020), CD4-FITC (#300506), CD8a-PerCP-Cy5.5 (#301032), CTLA4-PE (#369604), IgG2a-PE (#400214), KLRG1-PE-Dazzle (#367716), IgG2a- PE-Dazzle 594 (#400222), TIM3-PE-Cy7 (#345034), IgG1-PE-Cy7 (#400176), TIGIT-APC (#372714), IgG2a-APC (#400232), PD-1-BV421 (#329920), IgG1-BV421 (#400158), LAG3-BV650 (#369324), IgG1-BV650 (#400162), CD45RA-PE-Dazzle 594 (#304146), CD107a-PE-Cy7 (#328618), CD25-BV421 (#302629), FoxP3-Alexa647 (320113), IgG1-Alexa647 (#400135), CD45RO-BV605 (#304238), CD127-BV650 (#351326). All surface antibodies were used at 1:100 dilution and fixed with 1% formalin overnight before measurement. Intracellular staining of FoxP3 was performed at 1:50 dilution according to the manufacturer’s protocol (True Nucelar Transcription Factor Kit, Biolegend). Flow cytometric analysis was performed using the Cytoflex S cytometer from Beckman Coulter equipped with the Cytexpert Software (2.3). The gating strategy for immune cell population analysis can be found in the supplementals (Figs. S1 and 2). Tumor cells were gated as CD45^−^ stromal cells.

### Statistics

Statistical analysis was performed using SPSS 26.0 and GraphPad Prism 6. P values of less than 0.05 were considered significant. Unpaired, 2‑tailed Student’s *t* test was used to assess significance.

## Results

### Recruitment

**Fig. 1:**
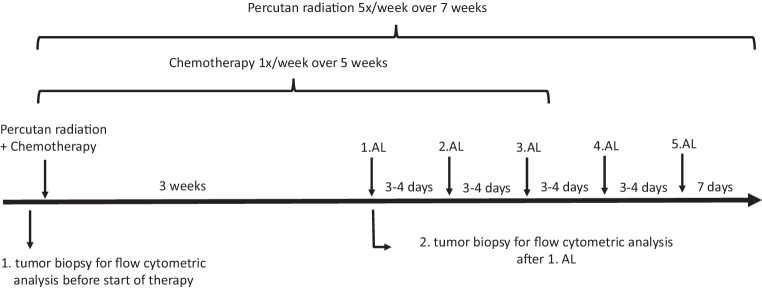
Course of radiochemotherapy for stage II and stage III cervical cancer patients. (*1.*–*5.AL* 1st to 5^th^ afterloading [Brachytherapy])

Between January 2020 and December 2021, patients with an indication for primary chemoradiation on FIGO stage II–III cervical cancer were recruited for the study. From all the participants, 34 were determined eligible for the study. However, 2 patients declined study participation and 10 patients were excluded because no tumor tissue was obtainable at the end of chemoradiation. Overall, 22 patients were analyzed. Basic clinical data are presented in Table 1 and a schematic of the study and biopsy time points are displayed in Fig. 1.

**Table 1:** Patients’ chararcteristics and treatment parameters

Characteristic	*n* = 22
Mean age—years	50.0 ± 13.2
*Stage*	
FIGO IIA	7 (27.3%)
FIGO IIB	8 (36.4%)
FIGO IIIA	1 (4.5%)
FIFO IIIB	7 (31.8%)
*N stage*	
pNx	2 (9.1%)
pN0	8 (36.4%)
pN1	12 (54.5%)
*Histology*	
Adenocarcinoma	4 (18.2%)
Squamous cell carcinoma	18 (81.8%)
*Laboratory values*	
Mean C‑reactive protein (first biopsy)—mg/L	8.74 ± 11.42
Mean C‑reactive protein (last biopsy)—mg/L	14.11 ± 20.47
Mean leukocytes (first biopsy)—10^3^/mL	8.19 ± 1.98
Mean leukocytes (last biopsy)—10^3^/mL	3.82 ± 1.53
*Chemotherapy*^a^	
Cisplatin	21 (95.5%)
Carboplatin	2 (9.1%)
*Target volumes*	
PTV (cm^3^) without para-aortal field	1340 ± 355
PTV (cm^3^) with para-aortal field	1643 ± 354
SIB (cm^3^)^b^	153 ± 65
Para-aortal field	7 (32%)

*pNx* lymph node status unknown, *pN0* negative lymph nodes, *pN1* lymph node metastases, *PTV* planning target volume (50.4 Gy); mean with SD

^a^ 1 patient was switched from cisplatin to carboplatin due to worsening renal function

^b^ 1 patient did not receive a simultaneous integrated boost (SIB)

### Chemoradiation depletes T cells but increases macrophage and neutrophil infiltration in the tumor

**Fig. 2:**
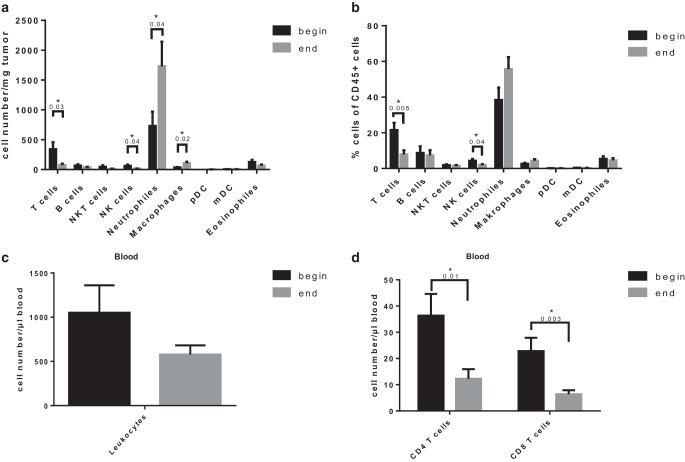
Tumor immune cell populations before and at the end of radiochemotherapy as determined by flow cytometry. **a** Tumor-infiltrating immune cell number/mg tumor. **b** Tumor-infiltrating immune cell distribution in % cells of CD45+ tumor cells. **c** Leukocytes in blood before and at the end of radiochemotherapy. **d** T cell populations in blood before and at the end of radiochemotherapy. Presented is mean ± SEM *p < 0.05, *n* = 22

To investigate the effect of chemoradiation on the tumor immune microenvironment, we analyzed a broad spectrum of immune cell populations in patient samples before and after chemoradiation. We observed a significant reduction of absolute T cells and natural killer cells (NK cells) in the tumor following chemoradiation (Fig. 2a, *p* 0.03 and 0.04, respectively), and a trend towards reduced numbers of B cells, natural killer T cells (NKT cells), plasmacytoid dendritic cells (pDC), myeloid dendritic cells (mDC) and eosinophils. Surprisingly and in contrast to other investigated cell populations, our results revealed that the number of neutrophils and macrophages significantly increased (Fig. 2a, *p* 0.04 and 0.02, respectively).

While percentages of T cells and NK cells were also significantly reduced in overall leukocytes infiltrating into tumors (Fig. 2b, *p* 0.01 and 0.005, respectively), the alterations for neutrophils and macrophages were less pronounced. Similar observations in the T cell compartment were made in the blood: While a trend towards decreased leukocytes was observed (Fig. 2c), percentages of CD4 and CD8 cells were significantly reduced following chemoradiation (*p* 0.01 and *p* 0.003 respectively, Fig. 2d).

We thus observed a general decrease of immune cells, in particular T cells, in both tumor and blood, and an increased infiltration of acute innate inflammatory cells such as macrophages and neutrophils at the end of chemoradiation compared to samples obtained before initiation of treatment.

### Chemoradiation significantly alters the CD8 inhibitory receptor profile and increases effector T cell populations

**Fig. 3:**
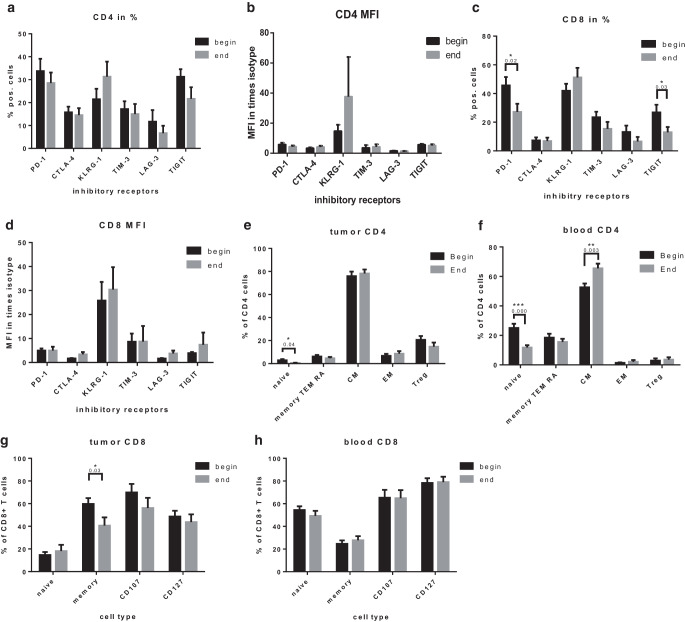
T cell characteristics before and at the end of radiochemotherapy. **a** Mean percentage of tumor-infiltrating CD4^+^ cells expressing the indicated inhibitory receptors. **b** Expression level on tumor-infiltrating CD4^+^ T cells expressing the indicated receptor normalized to fluorescence of isotype antibody. **c** Mean percentage of tumor-infiltrating CD8^+^ T cells expressing the indicated inhibitory receptors. **d** Expression level on tumor-infiltrating CD8^+^ T cells expressing the indicated receptor normalized to fluorescence of isotype antibody. **e**, **f** Mean percentage of naive (CD45RA^+^) and memory (CD45RO^+^), CD107^+^, CD127^+^ and FOXP3^+^, CD25^+^, CD4^+^ T cells in the tumor (**e**) and blood (**f**). **g**, **h** Mean percentage of naive (CD45RA^+^) and memory (CD45RO^+^), CD107^+^ and CD127^+^ CD8^+^ T cells in the tumor (**g**) and blood (**h**). Presented is mean ± SEM *p < 0.05, *n* = 22

Since tumor-infiltrating T cells are crucial for the anti-tumor response following immunotherapy, we next analyzed the T cell compartments of treated patients. Expression analysis of inhibitory T cell receptors on CD4 T cells did not reveal major changes in terms of abundance (Fig. 3a) or intensity (Fig. 3b). However, the frequency of PD‑1 and TIGIT-positive CD8 cells in tumors was significantly reduced (Fig. 3c, *p* 0.02 and 0.03, respectively), while expression intensity remained unaffected (Fig. 3d).

Further analysis revealed that naïve T cell populations significantly decreased in tumor-infiltrating CD4 cells (Fig. 3e, *p* 0.04) as well as in the blood (Fig. 3f, *p* 0.0002). Moreover, CD4 central memory T cell (T_CM_) populations increased significantly (*p* 0.003) while effector memory CD45 RA^+^ (T_EMRA_) and effector memory T cell (T_EM_) populations remained unchanged (Fig. 3f). In contrast to the CD4 compartment, CD8 T_CM_ were significantly reduced in tumors (*p* 0.03; Fig. 3g) but not in blood samples (Fig. 3h).

In summary, we observed an increase of CD4 T_CM_ in the blood, while naïve CD4 cells were diminished in both blood and tumors following chemoradiation. In the CD8 compartment, our results show a decrease in CD8 effector memory populations in the blood as well as decreased frequency of PD‑1 and TIGIT-positive CD8 cells in tumors.

### Chemoradiation decreases the frequency of PD-L1 but not PD-L2 expressing tumor cells

**Fig. 4:**
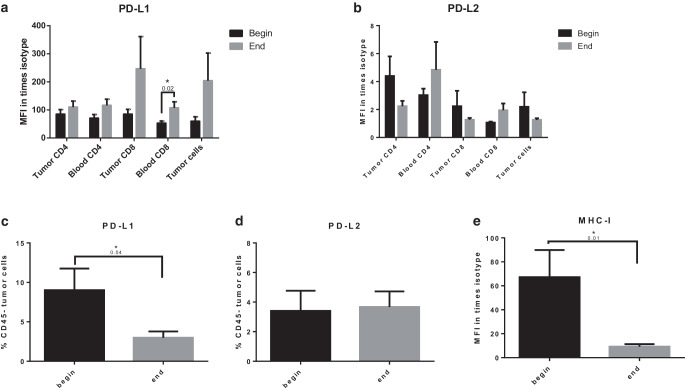
Expression of PD-L1 and PD-L2 on lymphocytes and tumor cells before and at the end of radiochemotherapy. **a** Expression intensity of PD-L1 on CD4^+^ and CD8^+^ T cells and CD45^−^ tumor cells normalized to isotype fluorescence. **b** Expression intensity of PD-L2 on CD4^+^ and CD8^+^ T cells and tumor cells normalized to isotype fluorescence. **c** Mean percentage of PD-L1 expressing CD45^−^ tumor cells. **d** Mean percentage of PD-L2 expressing CD45^−^ tumor cells. **e** MHC‑I expression on CD45^−^ tumor cells. Presented is mean ± SEM *p < 0.05, *n* = 22

Based on reports suggesting a predictive role of PD-L1 and PD-L2 expression on T cells in non-small cell lung cancer (NSCLC) [37], we also analyzed the expression of PD-L1 and PD-L2 on T cell in both blood and tumors in addition to tumor cells of patients before and at the end of chemoradiation. We found a robust expression intensity of PD-L1 and PD-L2 on CD8 and CD4 cells before and at the end of chemoradiation (Fig. 4a, b). Interestingly, PD-L1 but not PD-L2 expression seemed to be altered by chemoradiation on CD8 cells in the tumor: while there was a trend for increased PD-L1 on tumor CD8 cells (*p* 0.14), it was significantly upregulated on blood CD8 cells (*p* 0.02; Fig. 4a). Tumor CD4 cells showed a trend towards reduced expression intensity of PD-L2 (*p* 0.147) but not PD-L1 (Fig. 4a, b). Interestingly, while there was a trend towards increased PD-L1 levels on tumor cells (Fig. 4a, *p* 0.156), but not PD-L2 (Fig. 4b), the frequency of PD-L1-expressing tumor cells was significantly reduced at the end of chemoradiation (*p* 0.04; Fig. 4c). This was in contrast to the frequency of PD-L2-expressing cells, which remained unchanged (Fig. 4d). We also observed a significant decrease of MHC‑I expression intensity on tumor cells at the end of chemotherapy (Fig. 4e, *p* 0.01). Thus, we detected that while chemoradiation causes upregulation of PD-L1 on blood CD8 cells, it decreases the frequency of PD-L1-positive tumor cells and suppressed MHC‑I expression.

## Discussion

Using a detailed analysis of immune cell infiltrates before and after definitive chemoradiation of cervical cancer, we found that chemoradiation significantly alters both the CD8 inhibitory receptor profile and the PD-L1 profile on the tumor cells as well as the expression of the antigen presenting molecule MHC‑I.

While, to our knowledge, this is the first study to address the changes in the immune cell profile of patients undergoing definitive treatment, a similar analysis has been performed by Tsuchiya et al. in patients receiving pre-operative radiation treatment [38]. While this study similarly reported decreased numbers of T cells in tumors following treatment (a finding also supported by previous reports [39]), Tsuchiya et al. found that PD-L1 on tumor cells was increased [38]. In addition, Iijima et al. reported an upregulation of PD-L1 in cervical tumor cell lines and patient tumor samples after radiation treatment [40]. Both studies reported unchanged levels of PD-L1 expression on immune cells. However, their analysis was based solely on cell configuration and contrast hematoxylin and eosin (H&E) staining [38]. Another study investigating neoadjuvant radiation and chemoradiation in cervical cancer patients using immunohistochemistry does not address PD-L1 changes on tumors cells, but reports an overall stable expression pre- and posttherapy on lymphocytes (15 decrease or stable, 22 increased) [41]. In contrast, we found, using multiparametric flow cytometry, that PD-L1 expression intensity on both CD8 cells (while dramatically decreased in numbers after chemoradiation) and tumor cells showed a trend towards higher expression levels. Furthermore, due to the heterogenous nature of tumor cells and thereby lack of specific tumor markers, the tumor cells were identified as CD45 negative. Therefore, the discrepancy between this study and the previous ones might be attributed to the selection of a population consisting of tumor cells and other nonhematopoietic cells such as endothelial cells and fibroblasts. Another possible explanation on conflicting results is the sample analyzed: while the study of Tsuchiya et al. examined surgery specimen, i.e. expression at the time of surgery after neoadjuvant chemoradiation, and the study of Iijima et al. analyzed changes in cell lines after carbon-ion radiotherapy, our study investigated patient samples at the end of the chemoradiation regimen during treatment. However, our observation of decreased frequency of PD-L1 expressing cells in tumor samples remains unexpected, especially as radiation has been demonstrated to upregulate PD-L1 in other tumor entities both preclinically [42–45] and clinically [46].

RT therapy can lead to immune activation by release of damage-associated molecular patterns (DAMPs), IFN, activation of cGAS/STING pathway and MHC‑I upregulation. However, it can also lead to immune suppression by upregulation of PD-L1 and recruitment of immune suppressive cells like M2 macrophages or myeloid-derived suppressor cells (MDSC) [47]. Therefore, the addition of immune checkpoint inhibitors like PD-1/PD-L1 could counteract these immunosuppressive effects. Clinical studies suggest that PD-1/D-L1 blockade is beneficial in patients with PD-L1-positive tumors [48]. Recently, PD-L1 expression has been reported in patients with diverse tumor entities treated with RT only or in combination with chemotherapy [47]. However, other studies show conflicting results for patients receiving chemoradiation. A previous study detected unchanged expression of PD-L1 in the majority of rectal cancer patients, whereas another demonstrated downregulation of PD-L1 in NSCLC patients and indicated a poor prognosis for PD-L1-positive tumors [49]. These contradicting findings illustrate the complex relationship between PD-L1 and treatment benefits and indicate that RT might lead to immune evasion and even poorer prognosis. Therefore, it is essential to plan the administration and timing of anti-PD-1/PD-L1 treatment to chemoradiation cautiously.

Several studies have investigated immune cell infiltrates in cervical cancer and its prognostic value both in blood [50–52] and tumor [53–55]. Of particular interest appear to be both CD4, especially regulatory T cells (T_reg_ cells) [56], and CD8 cells [57]. In this light, it is interesting that while we found no change in T_reg_ cells percentages before and after treatment, we observed an enriched memory phenotype in the blood CD4 pool, a finding in line with previous reports [21], and alterations of inhibitory receptors. The first studies targeting TIGIT have recently been reported [58], and conventionally fractionated radiotherapy has been demonstrated to down-regulate TIGIT on CD8 cells in preclinical studies [59], in agreement with our results of decreased percentage of TIGIT-expressing cells in tumors. Although radiation is largely believed to increase PD‑1 in T cells [60, 61], our analyses, in line with Fujimoto et al., showed a decreased expression in CD8 cells in tumors [49].

Moreover, we observed an increase in neutrophils and macrophages in tumors following chemoradiation, which was unexpected considering previous reports of overall decreased immune cell infiltrates [44, 62]. This increase correlated with the elevated serum CRP levels, suggesting an inflammatory origin. Leukocytes, however, were decreased both in our blood analysis and in regular patient lab work, probably due to chemotherapy. In addition, the decrease of lymphocytes can be caused by radiation of the pelvis [63]. Blood lymphocytes reduction has been previously reported in patients undergoing chemoradiation for cervical cancer [64]. It remains unclear if this increase of tumor myeloid cells and serum CRP was due to radiation-induced inflammation or due to a susceptibility of localized infection. While clinical symptoms of inflammation such as itch, discharge, and discomfort are common during chemoradiation, no patient revealed clinical signs of infection upon inspection or on microbiological analysis, pointing to a radiation-induced inflammatory reaction as the origin of these symptoms rather than infection.

## Conclusion

Taken together, our study demonstrates that chemoradiation significantly affects the immune system in tumor and blood. Our results indicate a decline of inhibitory receptors on CD8 T cells, especially PD‑1, and a decreased expression of PD-L1 and MHC‑I on tumor cells after chemoradiation. These findings argue for neoadjuvant or simultaneous use of anti-PD‑1 or anti-PD-L1 treatment in upcoming clinical studies. Clinical studies with this regimen of immunotherapy are currently being conducted (CERAD IMMUNE, EudraCT number 2021-005208-36). However, the effect of single-dose, fractionation, target volume, and scheduling of external beam radiation and brachytherapy remains an open question.

## Supplementary Information

Supplemental figures S1 and S2
