# Supplementary material for: Influence of chemoradiation on the immune microenvironment of cervical cancer patients
Source: Strahlenther Onkol. 2022 Oct 17;199(2):121–30. doi: 10.1007/s00066-022-02007-z (PMC9876875; doi:10.1007/s00066-022-02007-z)
Supplement: Supplementary file 1 — Supplemental figures S1 and S2 [file 66_2022_2007_MOESM1_ESM.pdf]

Supplemental

A

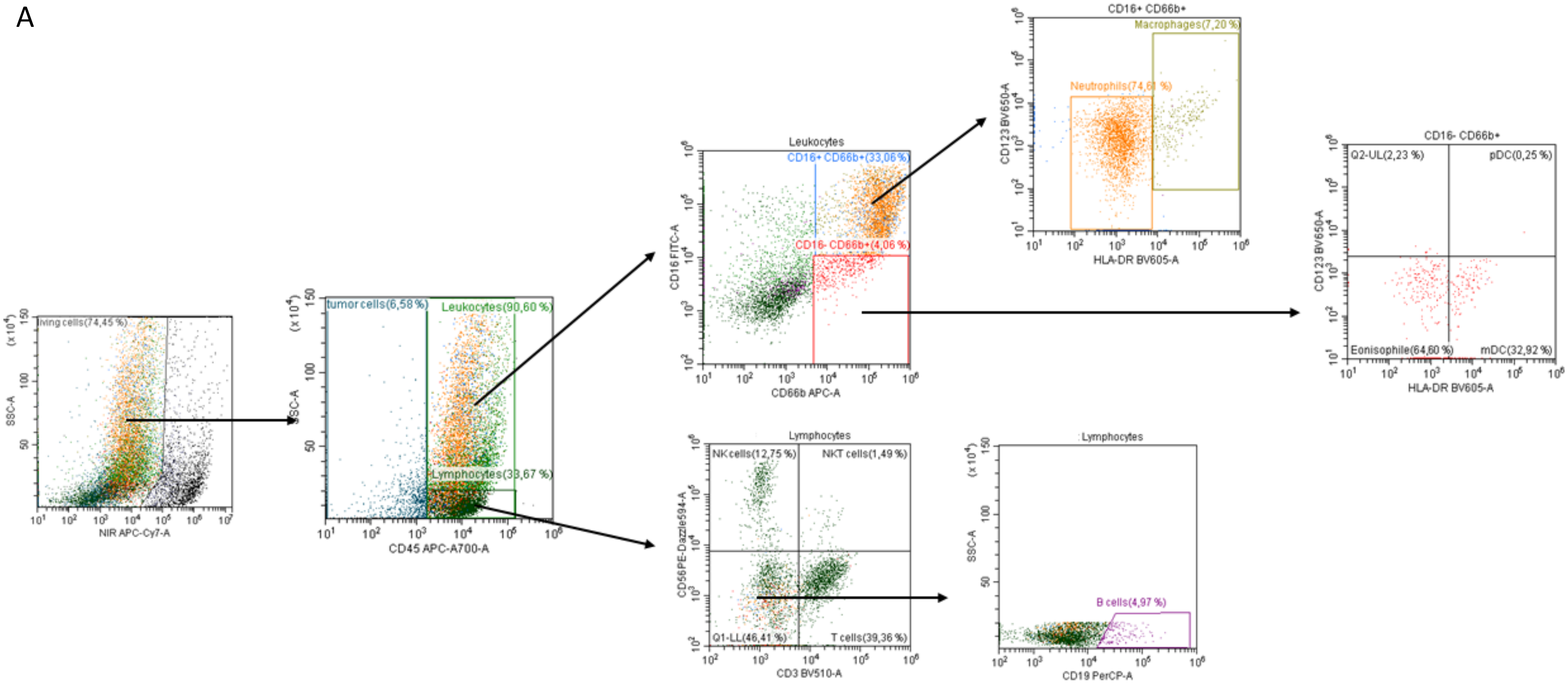

living cells(74,45 %)
SSC-A
NIR APC-Cy7-A
tumor cells(6,58 %)
Leukocytes(90,60 %)
Lymphocytes(33,67 %)
CD45 APC-A700-A
Leukocytes
CD16+ CD66b+(33,06 %)
CD16- CD66b+(4,06 %)
CD16 FITC-A
CD66b APC-A
CD16+ CD66b+
Macrophages(7,20 %)
CD123 BV650-A
HLA-DR BV605-A
CD16- CD66b+
Q2-UL(2,23 %)
pDC(0,25 %)
Eonisophile(64,60 %)
mDC(32,92 %)
Lymphocytes
NK cells(12,75 %)
NKT cells(1,49 %)
Q1-LL(46,41 %)
T cells(39,36 %)
CD56 PE-Dazzle594-A
CD3 BV510-A
B cells(4,97 %)
CD19 PerCP-A

B

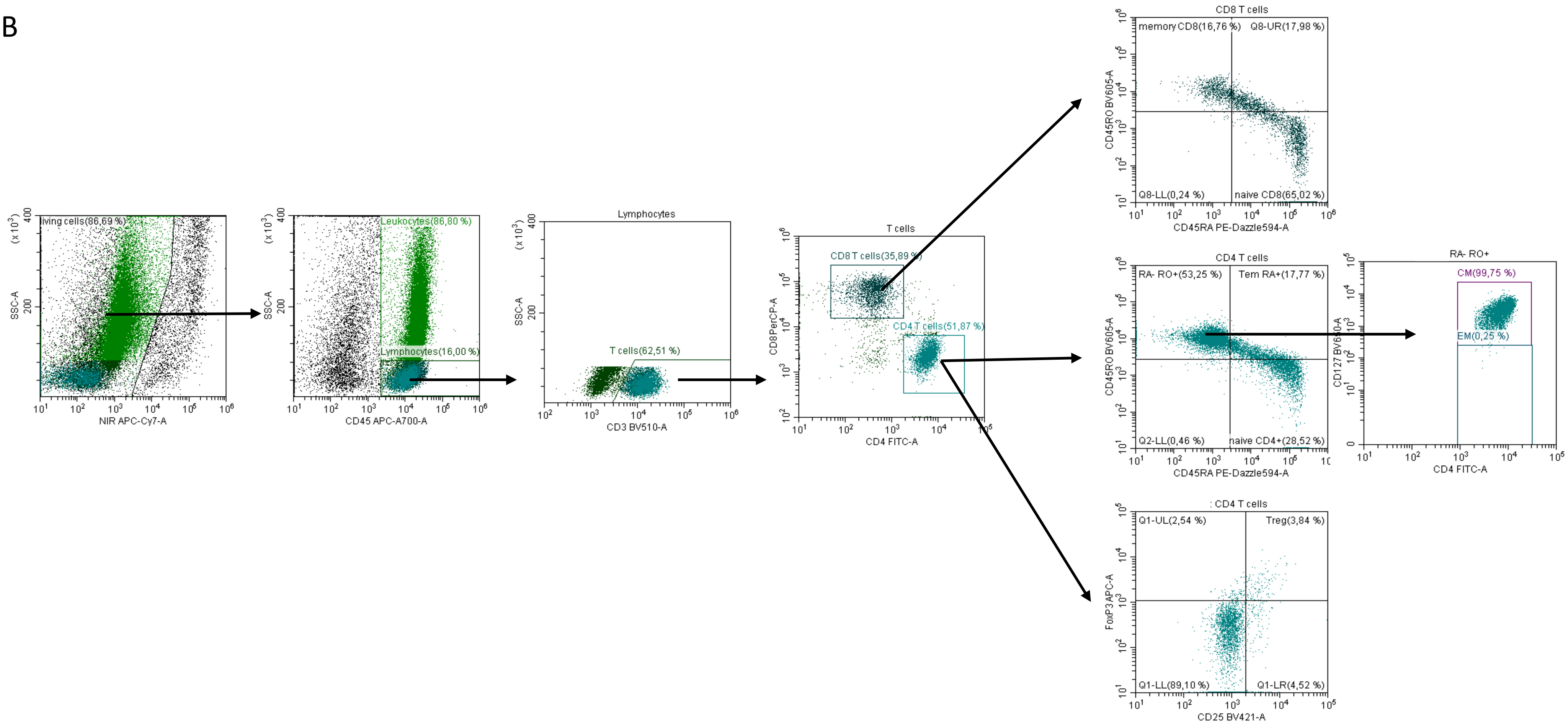

Supplemental figure S1: Gating strategy for flow cytometry

A. Gating strategy for innate immune cells, T cells and B cells. B. Gating strategy for CD4 and CD8 cells.

			I	II	III (Intracell)	VI
blue	488 nm	FITC	CD16	CD4	CD4	CD4
		PerCP-Cy5.5	CD19	CD8	CD8	CD8
yellow-green	561 nm	PE	PD-L1 (Iso)	CTLA-4 (Iso)	IFNy (Iso)	PD-L1 (Iso)
		PE-Dazzle594	CD56	KLRG1 (Iso)	CD45RA	
		PE-Cy5				
		PE-Cy7	MHCI	TIM3 (Iso)	CD107a (Iso)	
red	633 nm	APC	CD66b	TIGIT (Iso)	FoxP3 (Iso)	LFA1
		Alexa700	CD45	CD45	CD45	CD45
		APC-Cy7	NIR	NIR	NIR	NIR
violet	405 nm	BV421	PD-L2 (Iso)	PD-1 (Iso)	CD25	PD-L2 (Iso)
		BV510	CD3	CD3	CD3	CD3
		BV605	HLA-DR	LAG3 (Iso)	CD45RO	
		BV650	CD123		CD127	

tumor (I–VI)

blood (III–VI)

Supplemental figure S2: Flow cytometric antibody panel.
